# Phosphorylated Tau 181 Serum Levels Predict Alzheimer’s Disease in the Preclinical Stage

**DOI:** 10.3389/fnagi.2022.900773

**Published:** 2022-06-13

**Authors:** Wei Qin, Fangyu Li, Longfei Jia, Qi Wang, Ying Li, Yiping Wei, Yan Li, Hongmei Jin, Jianping Jia

**Affiliations:** ^1^Innovation Center for Neurological Disorders and Department of Neurology, Xuanwu Hospital, Capital Medical University, National Clinical Research Center for Geriatric Diseases, Beijing, China; ^2^Beijing Key Laboratory of Geriatric Cognitive Disorders, Capital Medical University, Beijing, China; ^3^Clinical Center for Neurodegenerative Disease and Memory Impairment, Capital Medical University, Beijing, China; ^4^Center of Alzheimer’s Disease, Beijing Institute of Brain Disorders, Collaborative Innovation Center for Brain Disorders, Capital Medical University, Beijing, China

**Keywords:** Alzheimer’s disease, preclinical stage, phosphorylated tau 181, serum, biomarker

## Abstract

**Background:**

There is an urgent need for cost-effective, easy-to-measure biomarkers to identify subjects who will develop Alzheimer’s disease (AD), especially at the pre-symptomatic stage. This stage can be determined in autosomal dominant AD (ADAD) which offers the opportunity to observe the dynamic biomarker changes during the life-course of AD stages. This study aimed to investigate serum biomarkers during different AD stages and potential novel protein biomarkers of presymptomatic AD.

**Methods:**

In the first stage, 32 individuals [20 mutation carriers including 10 with AD, and 10 with mild cognitive impairment (MCI), and 12 healthy controls] from ADAD families were analyzed. All subjects underwent a complete clinical evaluation and a comprehensive neuropsychological battery. Serum samples were collected from all subjects, and antibody arrays were used to analyze 170 proteins in these samples. The most promising biomarkers were identified during this screening and were then measured in serum samples of 12 subjects with pre-MCI and 20 controls.

**Results:**

The serum levels of 13 proteins were significantly different in patients with AD or MCI compared to controls. Of the 13 proteins, cathepsin D, immunoglobulin E, epidermal growth factor receptor (EGFR), matrix metalloproteinase-9 (MMP-9), von Willebrand factor (vWF), haptoglobin, and phosphorylated Tau-181 (p-Tau181) correlated with all cognitive measures (*R^2^* = −0.69–0.76). The areas under the receiver operating characteristic curve of these seven proteins were 0.71–0.93 for the classification of AD and 0.57–0.95 for the classification of MCI. Higher levels of p-Tau181 were found in the serum of pre-MCI subjects than in the serum of controls. The p-Tau181 serum level might detect AD before symptoms occur (area under the curve 0.85, sensitivity 75%, specificity 81.67%).

**Conclusions:**

A total of 13 serum proteins showed significant differences between subjects with AD and MCI and healthy controls. The p-Tau181 serum level might be a broadly available and cost-effective biomarker to identify individuals with preclinical AD and assess the severity of AD.

## Introduction

Alzheimer’s disease (AD) is the most common form of dementia among the elderly globally. Studies indicate that the brain pathology of AD starts to develop at least 10–20 years before the disease becomes clinically symptomatic (Bateman et al., 2012). This provides a window of opportunity to initiate preventive treatment. There is a need to identify widely available, easy-to-measure, and cost-effective biomarkers to identify AD in this pre-symptomatic stage (Molinuevo et al., 2018). Although autosomal dominant AD (ADAD) represents fewer than 1% of all AD cases, it provides a unique opportunity to investigate biomarker levels during this stage because the associated mutations are almost 100% fully penetrant, and symptom onset is relatively predictable in mutation carriers (Bateman et al., 2011; Sanchez-Valle et al., 2018).

Cerebrospinal fluid (CSF) biomarkers have shown strong correlations with clinical and cognitive measures in ADAD (Fagan et al., 2014). However, repeated CSF sampling is neither feasible nor cost-effective. The determination of serum biomarkers is less invasive, less costly, and can be performed more frequently than CSF investigations (Hampel et al., 2018; Zetterberg and Blennow, 2020).

Some studies have suggested that energy metabolism disorders, vascular alteration microenvironment hypoxia, oxidative stress, cell death, and chronic inflammation are also major contributors to the cognitive decline and neurodegenerative disorders associated with AD (Custodia et al., 2022; Yassine et al., 2022).

In this study, a custom protein chip was developed using 170 candidate biomarkers that have been implicated in AD. These proteins included synaptic proteins, inflammation factors, circulating cytokines, chemokines, and growth factors, et al. Then these protein levels were analyzed in serum samples from subjects in different stages of AD within the Chinese Familial Alzheimer’s Disease Network. We also examined the relationship between potential candidate biomarkers and cognitive function measures such as MMSE scores. We aimed to establish the characteristic serum protein profiles in different stages of AD and potential novel protein biomarkers of presymptomatic AD.

## Materials and Methods

### Study Design and Setting

All subjects of this study were selected from the Chinese Familial Alzheimer’s Disease Network (CFAN), which is a multicenter, longitudinal cohort of familial AD (Jia et al., 2020, 2021). They were consanguineous members of families with mutations in the genes encoding the amyloid-beta precursor protein (*APP*), presenilin-1 (*PSEN1*), or presenilin-2 (*PSEN2*, Swardfager et al., 2010). Subjects who carried the mutations were identified, and family members that did not carry a mutation served as controls. This retrospective study consisted of two stages. In the first stage, we analyzed 170 protein levels using an antibody array in serums from 10 AD subjects, 10 MCI subjects, and 12 controls. In the second stage, we selected some proteins and assessed their abilities to distinguish patients with pre-MCI from controls using ELISA. The selection criterion is: the significantly different levels of proteins between control and AD/MCI, significant associations with all cognitive measures, and moderate or high accuracy in predicting MCI and AD (AUC > 0.8, sensitivity > 80%, and specificity > 80%). Twelve pre-MCI subjects and 20 controls were included.

The study protocol was approved by the Ethics Committee of Xuanwu Hospital, Capital Medical University, and all subjects gave written informed consent.

### Clinical and Cognitive Assessment

The participants recruited in CFAN underwent a complete clinical evaluation, the tests of the known causative AD genes, and a comprehensive neuropsychological battery. The diagnosis of AD or MCI related to AD was made according to the National Institute on Aging and Alzheimer’s Association (NIA-AA) criteria (Albert et al., 2011; McKhann et al., 2011). Subjects were classified as having pre-MCI if they carried one of the mutations, had no cognitive complaints, and a normal cognitive performance. All MCI patients had a “high likelihood” of developing AD according to the NIA-AA criteria (i.e., meeting the core clinical criteria for MCI plus carrying autosomal dominant mutations; Albert et al., 2011). All AD patients were diagnosed with “probable AD dementia (probable AD dementia in a carrier of an ADAD genetic mutation; McKhann et al., 2011). The controls were cognitively normal and had neither amnesia nor did they carry mutations in genes related to AD.

The neuropsychological battery assessed the cognitive domains of verbal ability, visuospatial construction, episodic memory, and executive functions. Cognitive progression was measured using the Mini-Mental State Examination (MMSE), Montreal Cognitive Assessment (MoCA), activities of daily living (Newman et al., 2021) and Clinical Dementia Rating (CDR). Both the MMSE and the MoCA are routine cognitive screening tests rated on a 30-point scale. An MMSE score or a MoCA score below the cutoff was used to classify patients as having a cognitive impairment (lower scores indicating greater impairment; Chen et al., 2016; Li et al., 2016). The cutoff varies with different education levels. The score on the ADL ranges from 0 to 80, with a lower score indicating a better functional independency (Mlinac and Feng, 2016). The algorithm-generated global CDR score produces a total possible score of 0–3, connoting a global level of functional status from no cognitive impairment (CDRglobal 0) to severe impairment (CDRglobal 3; Morris, 1993). The CDR sum of boxes score (CDRsob), by contrast, utilizes a summary of the individual domain box scores and yields a total score of 0–18 (higher scores indicating greater impairment), and is frequently used in dementia staging and tracking of progression over time (O’Bryant et al., 2008).

### Genetic Screening

Serum samples analyzed in this study were obtained from this cohort CFAN. As a part of the routine assessment, genomic DNA was extracted from the peripheral blood samples as described previously (Qin et al., 2011). Exons 3–12 of the *PSEN1*, exons 1–12 of the *PSEN2*, and exons 16 and 17 of the *APP* genes were amplified using polymerase chain reaction (PCR) and specific primers (see Additional file 1, Supplementary Table S1) and determined by Sanger sequencing. AD and MCI patients were screened for mutations in the *PSEN1*, *PSEN2*, and *APP* genes, whereas other family members were screened for the mutation segregating in their family using Sanger sequencing to identify their mutation status. *Apolipoprotein E* (*APOE*) genotypes were also determined by Sanger sequencing.

### Antibody Arrays

We measured the relative concentrations of a total of 170 proteins (see Additional file 1, Supplementary Table S2) with antibody arrays (RayBiotech Inc., Peachtree Corners, GA, USA) according to the manufacturer’s instructions. Briefly, a custom glass-based antibody array targeting the 170 proteins of interest was built, 100 μl of diluted serum sample was added to each well, incubated overnight at 4°C, and then extensively washed. We then incubated the wells with biotin-conjugated antibodies specific to the different proteins. Membranes were developed with Alexa Fluor^®^ 555-conjugated streptavidin (Thermo Fisher Scientific, Waltham, MA, USA). The slides were scanned with 532 nm excitation and extracted using an InnoScan^®^ 300 Scanner (Innopsys, Carbonne, France).

### Enzyme-Linked Immunosorbent Assay

The selected proteins were further tested in samples of pre-MCI and control subjects using enzyme-linked immunosorbent assays in the second stage. The assay kit included Human Cathepsin D DuoSet (DY1014-05, R&D Systems, Inc., Minneapolis, MN, USA), von Willebrand factor (vWF; Human vWF-A2 DuoSet, DY2764-05, R&D Systems, Inc., Minneapolis, MN, USA), and (Human Tau pT181 ProQuantum, A46739, Invitrogen Corp., Carlsbad, CA, USA). All serum samples and kit components were equilibrated to room temperature before the assay, and the detection procedures were performed in accordance with the manufacturers’ instructions. For cathepsin D and vWF detection, the serum samples were diluted, added to separate wells, and incubated with a sealed plate. After conjugation and washing with buffer, substrate solutions were added, and the wells were incubated for 30 min. Finally, a stop solution (Invitrogen Corp.) was added to stop the reaction, and the optical density was measured at 450 nm. For P-tau181 detection, the antibody-conjugate mixture and diluted samples were added to assay wells. After mixing thoroughly, they were incubated overnight at 4°C. Then quantitative PCR reactions were performed on the StepOnePlus™ Real-Time PCR System (Thermo Fisher Scientific, Waltham, MA, USA). Concentrations were calculated according to standard curves. Standard samples containing the recombinant proteins, subjects’ serum samples, and empty controls were all assayed in duplicates to reduce variation.

### Functional Profiling of the Identified Proteins

Gene ontology (GO) enrichment analyses were conducted to assess the enrichment of these proteins in specific biological processes, molecular functions, and cell components. We used the Kyoto Encyclopedia of Genes and Genomes (KEGG) database to analyze the pathway enrichment of these proteins, and obtained an overview of their potential functional relevance affected by AD. Gene Ontology enrichment analysis, Kyoto Encyclopedia of Genes and Genomes (KEGG) analysis, and the protein-protein interaction network were conducted in Metascape^1^ (Zhou et al., 2019).

### Statistical Analysis

Expression data from the two filters per sample were normalized to the median expression of all 170 proteins, followed by *Z*-score transformation (Ray et al., 2007).

Differences in categorical data between the groups, such as sex, and *APOE* ε4 carrier distributions, were analyzed using the χ^2^ test. Differences in numerical data between the groups were evaluated using analysis of variance with Bonferroni *post-hoc* tests. Multiple linear regression analyses were used to assess potential associations between serum proteins and cognitive measures after adjusting for confounders. Receiver operating characteristic (ROC) curves were drawn by plotting the sensitivity against 1-specificity for different cut-off values. The area under the curve (AUC) was calculated for each. GraphPad Prism statistical software (version 8.1.1, GraphPad Software, San Diego, CA, USA) was used for analyses. Statistical significance was based on two-sided tests with an adjusted *P*-value < 0.05.

## Results

### Study Participants

Table 1 shows the demographic and clinical characteristics of the enrolled individuals. Among the 20 symptomatic mutation carriers, 10 fulfilled the criteria of AD and 10 of MCI. The 20 mutation carriers showed 13 different mutations (number of subjects): F105I (*n* = 2), G378E (*n* = 1), H163R (*n* = 3), L282V (*n* = 1), L392V (*n* = 1), M139V (*n* = 1), G111V (*n* = 1), M139L (*n* = 2), and P433S (*n* = 1) mutations in the *PSEN1* gene, R62H (*n* = 1) and V214L (*n* = 1) mutations in the *PSEN2* gene, and V717I (*n* = 4) and I716T (*n* = 1) mutations in the *APP* gene (see Additional file 1, Supplementary Table S3). The second stage included 12 pre-MCI mutation carriers, and 20 controls. Twelve pre-MCI participants were mutation carriers with a known causative mutation of AD, including six carrying *PSEN1* mutation, four carrying *APP* mutation, and two carrying *PSEN2* mutation. Twenty controls were healthy non-carrier family members. The frequency of the *APOE* ε4 allele was higher in the AD and MCI groups than in the controls. The demographic data showed expected diagnosis-related cognitive characteristics with respect to MMSE, MoCA, CDRsob, and CDRglobal scores.

**Table 1:** Demographic and clinical characteristics of subjects within the Alzheimer’s disease (AD), mild cognitive impairment (MCI), and control groups.

	**Cohort 1**				**Cohort 2**		
	**Control**	**MCI**	**AD**	***P* values**	**Control**	**pre-MCI**	***P* values**
*N* = 40	12	10	10	-	20	12	-
Age (years),	44.83	48.30	50.90	0.30	45.25	32.25	<0.01
mean (SD)	(12.95)	(5.14)	(5.67)		(11.51)	(5.26)	
Sex, M/F	6/6	9/1	4/6	0.05	12/8	4/8	0.27
MMSE,	29	24	12	<0.01	29	28	0.98
mean (SD)	(1.41)	(5.46)	(5.37)		(1.36)	(2.56)	
MoCA,	26	21	7	<0.01	27	26	0.37
mean (SD)	(3.01)	(4.10)	(3.92)		(2.93)	(2.37)	
CDR,	0	0.5	2	<0.01	0	0	-
mean (SD)		(0.24)	(0.94)				
CDR-SOB,	0	1.85	10.05	<0.01	0	0.06	0.17
mean (SD)		(1.83)	(5.25)			(0.18)	
APOE ε4	2	2	4	0.41	3	2	0.90
carrier	(16%)	(20%)	(40%)		(15%)	(17%)	

*MMSE, Mini-Mental State Examination; MoCA, Montreal cognitive assessment; ADL, activities of daily living; CDR, Clinical Dementia Rating; CDRsob, Clinical Dementia Rating sum of boxes; SD, standard deviation*.

### Serum Proteins in Different Diagnostic Groups

**Figure 1:**
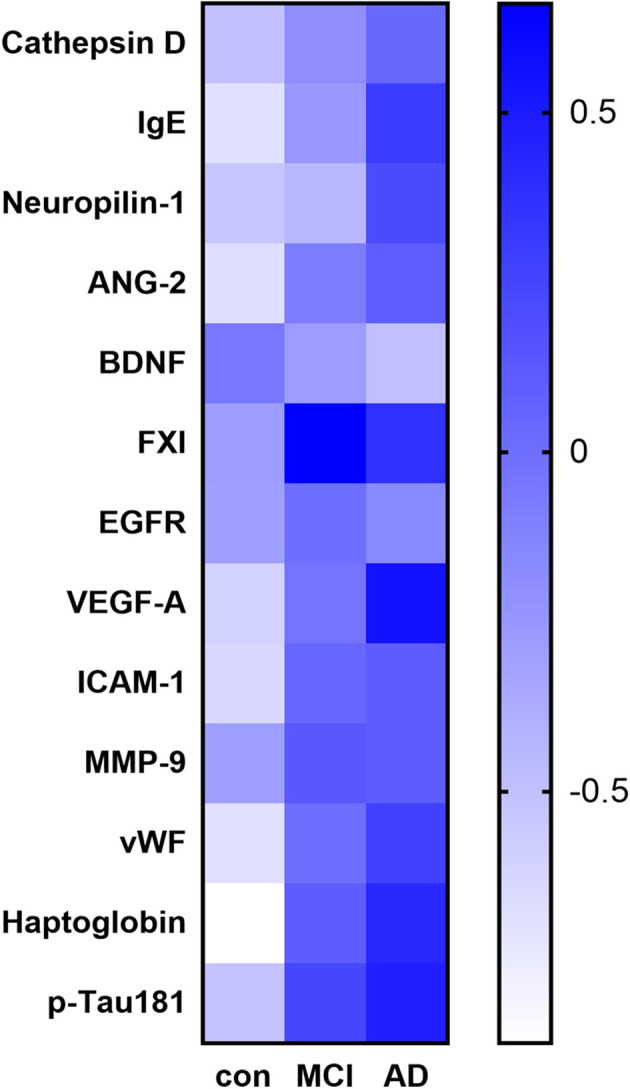
Heat map of the identified potential biomarker proteins. The heatmap shows the overall expression of the 13 proteins with a significant difference in their serum levels (adjusted *P* < 0.05) between subjects with Alzheimer’s disease (AD) or mild cognitive impairment (MCI) and the controls.

**Figure 2:**
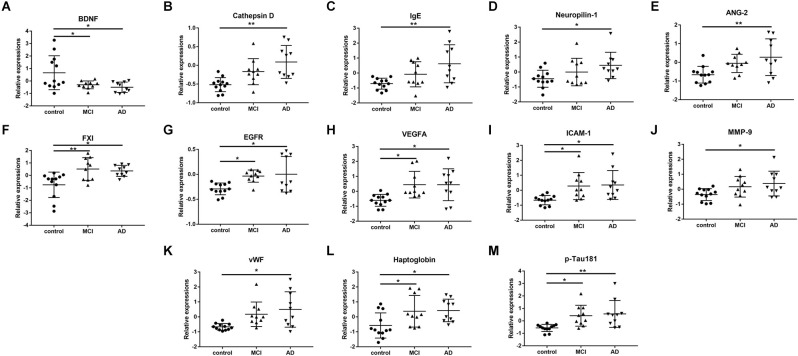
The scatter plots showed the detail comparisons of serum levels of identified potential biomarker proteins between the control, mild cognitive impairment (MCI), and Alzheimer’s disease (AD) groups.** (A)** Brain derived neurotrophic factor (BDNF); **(B)** Cathepsin D; **(C)** Immunoglobulin E (Chen et al., 2020); **(D)** Neuropilin-1; **(E)** Angiopoietin-2 (ANG-2); **(F)** Coagulation factor XI (FXI); **(G)** Epidermal growth factor receptor (EGFR); **(H)** Vascular endothelial growth factor A (VEGFA); **(I)** Intercellular adhesion molecule -1 (ICAM-1); **(J)** Matrix metalloproteinase-9 (MMP-9); **(K)** von Willebrand factor (vWF); **(L)** Haptoglobin; **(M)** Phosphorylated Tau-181 (p-Tau181). *P < 0.05, **P < 0.01.

Among the 170 proteins analyzed, we identified 13 proteins that were differentially expressed in the three groups after adjusting for gender and age (Figure 1). Brain-derived neurotrophic factor (BDNF) levels were significantly downregulated in the serum of MCI and AD patients compared to those in the controls. Significant higher cathepsin D, immunoglobulin E (Chen et al., 2020), neuropilin-1, angiopoietin-2 (ANG-2), coagulation factor XI (FXI), epidermal growth factor receptor (EGFR), vascular endothelial growth factor A (VEGFA), intercellular adhesion molecule 1 (ICAM-1), matrix metalloproteinase-9 (MMP-9), von Willebrand factor (vWF), haptoglobin, and p-Tau181 levels were found in the serum samples of AD subjects than in the samples of controls (Figure 2). FXI, EGFR, VEGFA, ICAM-1, haptoglobin, and p-Tau181 levels were also significantly different in the MCI vs. control group comparison (Figure 2). No significant differences in these protein levels were observed between MCI and AD subjects.

### Serum Proteins and Clinical Cognition

**Figure 3:**
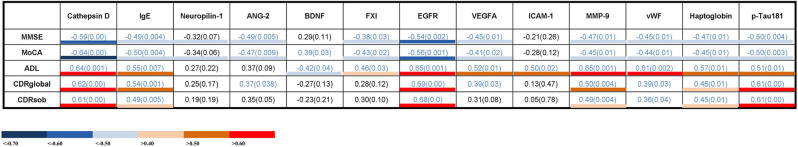
Correlations between serum levels of potential biomarker proteins and cognitive measures. The values presented are Spearman’s rank coefficients r^2^. Blue values indicate a *P* < 0.05. The color key indicates the strength of correlations based on the correlation coefficients. IgE, immunoglobulin E; ANG-2, angiopoietin-2; BDNF, brain-derived neurotrophic factor; EGFR, epidermal growth factor receptor; VEGF, vascular endothelial growth factor; ICAM-1, intercellular adhesion molecule -1; MMP-9, matrix metalloproteinase-9; vWF, von Willebrand factor; p-Tau, Phosphorylated Tau; MMSE, Mini-Mental State Examination; MoCA, Montreal cognitive assessment; ADL, activities of daily living; CDRglobal, Clinical Dementia Rating global; CDRsob, Clinical Dementia Rating sum of boxes.

The correlations between serum proteins and cognitive measures are shown in Figure 3. Seven of the thirteen proteins were significantly correlated with all cognitive measures. Cathepsin D, IgE, EGFR, MMP-9, vWF, haptoglobin, and p-Tau181 showed a negative correlation with the Mini-Mental State Examination (MMSE; *R^2^* = −0.59–0.45) and the Montreal cognitive assessment (MoCA) scores (*R^2^* = −0.64–0.44), and positive correlations with the activities of daily living (Newman et al., 2021; *R^2^* = 0.50–0.65), CDRglobal (*R^2^* = 0.39–0.69) or the CDR sum of boxes (CDRsob) scores (*R^2^* = 0.36–0.61). The higher serum Cathepsin D, IgE, EGFR, MMP-9, vWF, haptoglobin, and p-Tau181 levels were associated with severity of memory impairment (as indicated by lower MMSE and MoCA scores, and higher ADL, CDRglobal, and CDRsob scores). These results suggest a possible link between these serum proteins and cognitive decline.

### Functional Profiling of the Identified Serum Proteins

**Figure 4:**
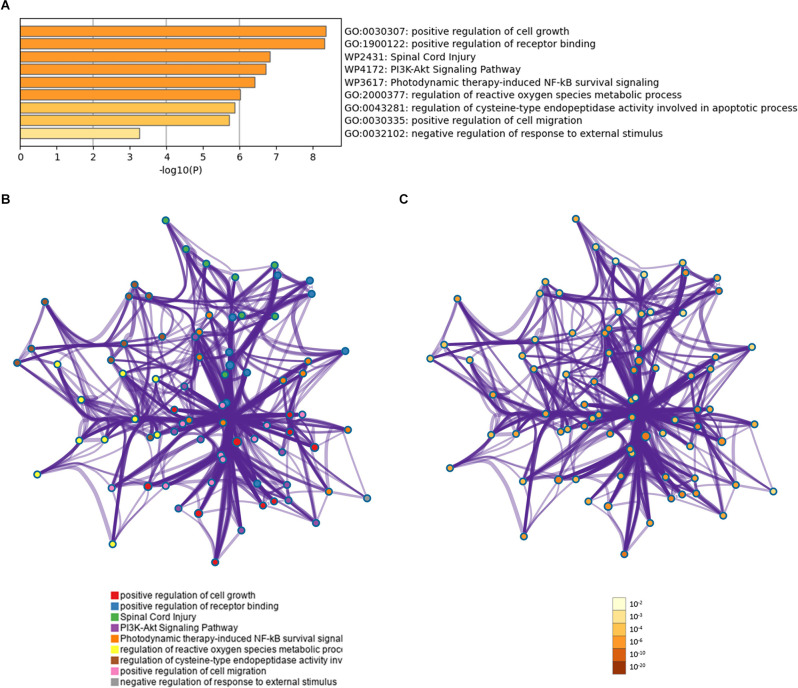
Functional profiling of the identified proteins. **(A)** Shown are significantly enriched gene ontology (GO) and Kyoto Encyclopedia of Genes and Genomes (KEGG) terms. **(B)** Protein-protein interaction network of GO and KEGG enriched terms colored according to clusters. **(C)** Protein-protein interaction network of GO and KEGG enriched terms colored according to *P*-values.

Gene ontology analyses indicated an involvement of the 13 proteins in the regulation of cell growth and the regulation of receptor binding (Figure 4A). The overall effect of up- or downregulation of the signaling proteins in the KEGG pathways predicted that the PI3K-Akt and NF-κB signaling pathway is involved in AD (Figure 4A). Network of GO and KEGG enriched terms colored according to clusters and *P*-values were also shown (Figures 4B,C).

### Predictive Value of Serum Proteins

In the ROC analysis, the identified 13 serum proteins showed moderately high or high AUCs for distinguishing subjects with AD or MCI from the controls. Sensitivity, specificity, accuracy, and the 95% confidence intervals are shown in Table 2. Cathepsin D, VEGFA, ICAM-1, vWF, and p-Tau181 were predicted both in MCI and AD with AUC values, sensitivity, and specificity above 0.8. Among them, cathepsin D had the highest AUC value for distinguishing AD from control (AUC=0.93), and its performance for discriminating MCI from control was moderate (AUC=0.84). The AUCs of p-Tau181 in differentiating AD or MCI from control were 0.89 and 0.91, respectively.

**Table 2:** Outcomes of the receiver operating characteristic curve analysis for the identified proteins.

	**MCI vs. control**					**AD vs. control**				
**Proteins**	**AUC**	**P value**	**Sensitivity**	**Specificity**	**95% CI**	**AUC**	**P value**	**Sensitivity**	**Specificity**	**95% CI**
Cathepsin D	0.84	0.007	80%	83.33%	0.66–1.02	0.93	0.0008	90%	83.33%	0.82–1.03
IgE	0.73	0.07	50%	100%	0.50–0.95	0.85	0.0056	70%	100%	0.68–1.03
Neuropilin-1	0.57	0.59	50%	66.67%	0.30–0.83	0.83	0.0084	80%	83.33%	0.66–1.00
ANG-2	0.82	0.01	80%	91.67%	0.62–1.01	0.78	0.025	70%	91.67%	0.58–0.99
BDNF	0.69	0.13	100%	41.67%	0.47–0.92	0.75	0.04	100%	41.67%	0.54–0.96
FXI	0.75	0.04	60%	100%	0.53–0.97	0.90	0.002	80%	91.67%	0.77–1.03
EGFR	0.94	0.0005	90%	100%	0.83–1.06	0.71	0.10	60%	83.33%	0.77–1.03
VEGF-A	0.93	0.0008	90%	83.33%	0.82–1.04	0.82	0.01	80%	91.67%	0.60–1.03
ICAM-1	0.88	0.002	80%	83.33%	0.74–1.03	0.92	0.001	80%	91.67%	0.80–1.03
MMP-9	0.73	0.06	60%	83.33%	0.50–0.96	0.77	0.03	60%	91.67%	0.56–0.98
vWF	0.92	0.001	90%	91.67%	0.79–1.04	0.82	0.01	70%	91.67%	0.60–1.02
Haptoglobin	0.80	0.02	100%	66.67%	0.61–0.99	0.83	0.01	100%	66.67%	0.66–1.00
p-Tau-181	0.91	0.001	80%	100%	0.78–1.04	0.89	0.002	80%	91.67%	0.75–1.03

*AUC, area under the curve*.

### Predictive Value of Serum Proteins for Pre-MCI

**Figure 5:**
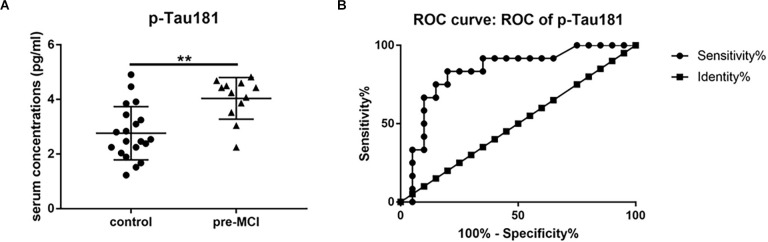
The serum levels and receiver operating characteristics (ROC) analysis of p-Tau181. **(A)** Serum levels of p-Tau181 in pre-symptomatic mild cognitive impairment (pre-MCI), and controls with black horizontal lines indicating median values. *P*-values were determined by analysis of variance with Bonferroni *post-hoc* tests, **P < 0.01. **(B)** ROC curve analyses of p-Tau-181 in pre-MCI vs. controls.

Cathepsin D, vWF, and p-Tau181 showed significant associations with cognitive measures and high accuracy in predicting MCI and AD. So we detected these three serum proteins in patients with pre-MCI and controls using ELISA. Among them, only serum p-Tau181 levels were statistically significantly higher in the pre-MCI subjects than in controls (2.76 pg/ml vs. 4.04 pg/ml, *P* < 0.01; Figure 5A). The ROC analysis showed that p-Tau181 had a high AUC for distinguishing pre-MCI subjects from controls (AUC 0.83, sensitivity 83.33%, specificity 80%; Figure 5B). These results indicate that the serum p-Tau181 has value in discriminating early stages of AD from healthy subjects.

## Discussion

In this study on potential biomarkers for the development of AD, we found that the serum levels of 13 proteins were significantly different in MCI and AD subjects from those in the controls, and seven of these proteins were correlated with cognitive measures. Among these, only serum p-Tau levels were higher in pre-MCI than in control subjects and were able to distinguish pre-MCI.

Detecting AD as early as possible is vital to enable trials of disease-modifying agents that aim to prevent the development of symptoms in individuals who are still cognitively normal. ADAD makes it possible to identify presymptomatic individuals decades before they are destined to develop clinical symptoms (Dubois et al., 2016). The ability to detect multiple analytes in a serum sample has encouraged further research of this screening method that is less invasive than CSF sampling.

Therefore, this study examined a total of 170 candidate serum biomarkers using samples of ADAD family members in an attempt to identify a cost-effective, rapid, and reliable biomarker for early AD. We found that both MCI and AD subjects showed lower BDNF serum levels and higher cathepsin D, IgE, neuropilin-1, ANG-2, FXI, EGFR, VEGFA, ICAM-1, MMP-9, vWF, haptoglobin, and p-Tau181 serum levels than controls. Seven of them significantly correlated with the MMSE, MoCA, ADL, and/or CDR scores. Cathepsin D, vWF, and p-Tau181 showed significant associations with cognitive measures and high accuracy in predicting MCI and AD. In the second stage, these three serum proteins were detected in patients with pre-MCI and controls using ELISA. An antibody array can quantify 170 different biomarkers simultaneously, so we used a custom antibody array to screen many biomarkers in the first stage. After selecting three candidate biomarkers, we further verified them using ELISA, which can detect one biomarker at a time in more samples.

Our result is consistent with a random-effects meta-analysis that showed that patients with AD had significantly lower baseline peripheral blood serum levels of BDNF compared with healthy controls (Qin et al., 2017). BDNF single-nucleotide polymorphism modulates the association between beta-amyloid (Aβ) and hippocampal disconnection in AD and is an important factor in cognitive impairment in AD (Franzmeier et al., 2021). Elevating BDNF levels improved cognition in an AD mouse model (Choi et al., 2018).

Notably, our study identified high serum levels of the hemostasis factors FXI and vWF in AD subjects, and these have previously been reported as potential AD biomarkers (Loures et al., 2019; Begic et al., 2020). We also found that higher levels of FXI and vWF were associated with lower MMSE and MoCA scores, and associated with higher ADL scores. The vWF showed an AUC of 0.92 and 0.82 when it was used to distinguish MCI or AD from controls, respectively. Previous studies also showed significantly higher FXI and vWF levels in AD patients compared to control subjects (Laske et al., 2011; Begic et al., 2020). Further, an increase in FXI was associated with a reduction in cognitive function in individuals. Impaired clot initiation and formation rates were found in the plasma of AD patients (Suidan et al., 2018). Ryu and McLarnon (2009) have demonstrated abnormal immunostaining of vWF in the brains of AD patients. These data suggest that biological pathways involving coagulation and anticoagulation factors are related to AD.

We found high serum levels of neuropilin-1, ANG-2, and VEGFA in MCI and AD patients, and VEGFA showed a 90% sensitivity and 83.33% specificity in predicting MCI. These three proteins are regulators of angiogenesis. Both ANG-2 and VEGFA were inversely correlated with the MMSE and MoCA scores. VEGFA is a pro-angiogenic factor that is essential during all stages of angiogenesis (Bosseboeuf and Raimondi, 2020). It can interact with the transmembrane protein neuropilin-1 to promote downstream signals, which are required for sprouting angiogenesis (Mamluk et al., 2002). Neuropilin-1 can also promote angiogenesis *via* VEGF-independent mechanisms and plays a role in regulating mitochondrial function and iron homeostasis, processes that are involved in the pathogenesis of AD (Kukreja et al., 2014; Peters et al., 2015). Muche et al. (2015) found up-regulation of VEGFA and neuropilin-1 in the entorhinal cortex with Aβ deposition in the Tg2576 mouse model. A clinical study suggested that neuropilin-1 modified the risk for poor cognitive scores based on APOE-ε4 status (Moore et al., 2020). ANG-2 has also been reported as upregulated in AD patients (Thirumangalakudi et al., 2006; Rocha de Paula et al., 2011).

An increasing number of studies reported that the brain in AD patients shows signs of inflammation (Janelidze et al., 2018; Park et al., 2020). We found a systemic inflammatory response in AD subjects, shown in the elevated serum levels of haptoglobin, IgE, and ICAM-1. Haptoglobin and IgE were negatively linked with MMSE and MoCA scores and positively linked with ADL and CDR scores. ICAM-1was able to distinguish AD patients from controls, with an AUC of 0.92, a sensitivity of 80%, and a specificity of 91.67%. Previous studies found increased plasma and brain haptoglobin levels in AD patients compared to controls (Song et al., 2015; Philbert et al., 2021) and an association between haptoglobin levels and the severity of cognitive impairment (Zhu et al., 2018). ICAM-1 level was higher in preclinical, prodromal, and dementia stages of AD (Janelidze et al., 2018) and linked with CDR-SB scores (Drake et al., 2021). However, Kester et al. (2011) did not find that ICAM-1 levels were significantly changed in AD. This conflicting result may be due to a different study population in terms of AD severity or a different course of AD or different kinds of test samples. In previous studies, haptoglobin suppressed amyloid fibril formation and prevented Aβ toxicity (Yerbury et al., 2009; Yerbury and Wilson, 2010). Haptoglobin and ICAM-1 levels have been suggested as useful markers of the progressive course of AD (Wang et al., 2015; Park et al., 2020). Allergy is a highly prevalent chronic inflammatory condition. Allergic mice with increased brain levels of IgE were found to have higher Tau phosphorylation in the brain (Sarlus et al., 2012). Our findings support the theory that inflammatory reactions underpin AD development and progression.

The MMP-9 was upregulated in the AD subjects in our study, which is consistent with other reports (Bruno et al., 2009; Gu et al., 2020). MMP-9 is a proteolytic enzyme that is critical for tissue formation, neuronal network remodeling, and blood-brain barrier integrity (Rempe et al., 2016; Ringland et al., 2020). A number of studies have shown that MMP-9 can influence AD pathogenesis and cognitive dysfunction through several mechanisms, including blood-brain barrier alterations, lipoprotein receptor shedding, inflammation, and neurodegeneration (Mroczko et al., 2013; Halliday et al., 2016; Shackleton et al., 2019).

Notably, we found altered levels of cathepsin D and EGFR in AD patients. Cathepsin D showed a sensitivity of 90% and specificity of 83.33% in identifying AD subjects. Both proteins are involved in autolysosomal functions, which contribute to the pathogenesis of AD (Uddin et al., 2019; Gadhave et al., 2021). In adult brains, pathological conditions such as AD activate EGFR in both neurons and astrocytes (Ceyzeriat et al., 2018). Polymorphisms in EGFR and cathepsin D genes have been associated with AD (Paz et al., 2015; Chen et al., 2018). Several studies have demonstrated that EGFR inhibitors may improve pathological and behavioral conditions in AD (Wang et al., 2013, 2017). They exert their therapeutic effects through the induction of autophagy and attenuation of reactive astrocytes (Tavassoly et al., 2021). Studies showed that the lysosome proteins are more sensitive to cellular metabolic alteration in AD compared to levels of Aβ or Tau proteins (Morena et al., 2017). Significantly higher levels of cathepsin D were found in patients with AD than in patients with frontotemporal dementia and healthy controls (Goetzl et al., 2015; Cheng et al., 2018). Previous findings support lysosomal enzymes as peripheral molecules that vary with the progression of AD, which makes them useful in recognizing preclinical AD (Goetzl et al., 2015; Morena et al., 2017).

Tau is a microtubule-binding protein that is increased and phosphorylated in AD and constitutes the main component in AD tangle and neurite pathology. Total Tau and p-Tau181 isoform levels were significantly increased in the CSF of AD patients (Dubois et al., 2014; Molinuevo et al., 2018; Zou et al., 2020). Karikari et al. (2020) found high p-Tau181 plasma levels in patients with AD and in MCI patients that developed AD. Studies (Barthelemy et al., 2020; Suarez-Calvet et al., 2020) showed that p-Tau181 levels were significantly increased in preclinical AD, when only subtle signs of Aβ pathology can be detected or as early as two decades before the development of aggregated tau pathology. Several studies indicated that p-Tau181 blood levels could accurately distinguish AD patients from other tauopathies in symptomatic AD (Janelidze et al., 2020; Thijssen et al., 2020). Consistent with these previous studies, we demonstrated that serum p-Tau181 levels were significantly higher in MCI and AD subjects than in controls and associated with all cognitive tests. Our data indicated that these serum proteins might be used to predict cognitive decline. Furthermore, we found that exclusively p-Tau181 serum levels were able to distinguish pre-MCI from controls. In Suárez-Calvet’s study (Suarez-Calvet et al., 2020), they measured blood p-Tau181 changes in the preclinical stage of sporadic AD. The stage of AD is determined by the cutoff of CSF and PET biomarkers. The results may vary across different cutoffs. While in our study, the AD causative gene mutation carriers who had a normal cognitive performance were used as preclinical subjects. The certainty of disease and predictability of symptom onset of AD enables to accurately identify AD in the pre-symptomatic stage. Barthelemy et al. (2020) also quantified the phosphorylation state of the tau protein in dominantly inherited AD. But they detected p-Tau levels in CSF. To the best of our knowledge, our study is the first one to investigate serum p-Tau181 in the preclinical ADAD. In summary, serum p-Tau181 may help in the prediction of AD before the onset of cognitive impairment.

### Limitations

This study had several limitations. First, the sample size was small. Because of the rarity of ADAD, the sample size limits the interpretation of our results that needs to be further explored in larger cohorts. Also, the association between serum proteins level and genetic mutations could not be analyzed because of the small sample size. Second, there are no ADAD data for all the proteins we analyzed, which precluded the comparison with published data. Third, this is a cross-sectional study. The shortness of the longitudinal evaluations of the individuals limits the interpretation of these results. We trust that future larger prospective studies investigating these serum biomarkers with a long-term follow-up will address these limitations.

## Conclusions

In summary, a total of 13 serum proteins showed significant differences between subjects with AD and MCI and healthy controls. Furthermore, the serum levels of ANG-2, VEGFA, haptoglobin, and p-Tau181 were correlated with cognitive impairment. We highlight that the serum p-Tau 181 was found to distinguish pre-MCI subjects from normal controls. It will be helpful for early AD diagnosis and high-risk population screening of AD and initiate preventive treatment in asymptomatic people with AD.

## Data Availability Statement

The datasets presented in this study can be found in online repositories. The names of the repository/repositories and accession number(s) can be found in the article/Supplementary Material.

## Ethics Statement

The studies involving human participants were reviewed and approved by the Ethics Committee of Xuanwu Hospital, Capital Medical University. The patients/participants provided their written informed consent to participate in this study.

## Author Contributions

WQ designed the project, performed the experiments, wrote and edited the manuscript. FL analyzed and interpreted data. LJ examined the patients. QW conducted genetic screening. YiL extracted DNA samples. YW and YaL helped to detect serum protein levels. HJ performed cognitive tests on participants. JJ designed the study and edited the manuscript. All authors contributed to the article and approved the submitted version.

## Conflict of Interest

The authors declare that the research was conducted in the absence of any commercial or financial relationships that could be construed as a potential conflict of interest.

## Publisher’s Note

All claims expressed in this article are solely those of the authors and do not necessarily represent those of their affiliated organizations, or those of the publisher, the editors and the reviewers. Any product that may be evaluated in this article, or claim that may be made by its manufacturer, is not guaranteed or endorsed by the publisher.

## Footnotes

^1^ http://metascape.org/
