# Supplementary material for: Phosphorylated Tau 181 Serum Levels Predict Alzheimer’s Disease in the Preclinical Stage
Source: Front Aging Neurosci. 2022 Jun 13;14:900773. doi: 10.3389/fnagi.2022.900773 (PMC9234327; doi:10.3389/fnagi.2022.900773)
Supplement: Supplementary file 1 [file Data_Sheet_1.pdf]

Table S1 The primers for sequencing *PSEN1, PSEN2* and *APP*

Primer	Sequence (5'to 3')
PS1-3F	ACTAACAATGGATGACCTGGTGAAATC
PS1-3R	GAGATGATAAGTGAATCCAGTCTGGCA
PS1-4F	CGTTACCTTGATTCTGCTGAGAATCTG
PS1-4R	TGCAGAGGCCTTCAAGGTGATGATGAC
PS1-5F	GTGGTAATGTGGTTGGTGAT
PS1-5R	CCCAACCATAAGAAGAACAG
PS1-6F	TCTGTACTTTTTAAGGGTTGT
PS1-6R	ACTTCAGAGTAATTCATCAACA
PS1-7F	GGAGCCATCACATTATTCTA
PS1-7R	GAGATGAGGAAAGAAAACAC
PS1-8F	CACCCATTTACAAGTTTAGC
PS1-8R	GATGAGAACAAGTACCATGAA
PS1-9F	TGGCTTGTTGTTGTCTATGC
PS1-9R	AAGACGATAAAAACATTGCT
PS1-10F	AACTTCCACTTTCTCTTGAA
PS1-10R	GTAGCTACCTAAAGGAATCC
PS1-11F	AAGAGTGACCAACTTTTTAAT
PS1-11R	GTGTGGCCAGGGTAGAGAACT
PS1-12F	GTGATTGAGTTTTGCCTGAA
PS1-12R	TAGTCAAAGAAGAAACATCC
PS2-1F	TGTTAGCAGCGGTGTTTG
PS2-1R	TCTGCTCGGAGGGATGGAC
PS2-2F	CAGGGCCAGGGGGAGGAA
PS2-2R	AAAAGCAGGTTGGGAGTCAC
PS2-3F	GTCCTCCACTGCCTTTGTCTCAC
PS2-3R	CTTCCCTTCTCCCTCCCGCATCAG
PS2-4F	AAAAATCCGTGCATTACAT
PS2-4R	GCTGGTTGTGAGCTGCAGGTACAGTG
PS2-5F	AGCCTCGAGGAGCAGTCAG
PS2-5R	GCAGACGGAGAGAAGCGT
PS2-6F	GGTATCAGTCTCAGGATCATGGG
PS2-6R	TGGGGAAGACTGGAGCTCGATG
PS2-7F	GTAAAGAGGGCCAGGTTGGG
PS2-7R	GTGCAGCACTGGGGACGATTT
PS2-8F	GGGCAGGCTCTTCTTCAGGG
PS2-8R	GAAAGCCACGGCCAGGAAG
PS2-9F	ACCGCCTGAGACGTGAACCTT
PS2-9R	TCCCTCTGCCCCTCCTGAACT
PS2-10F	CTCTGACCAGCTGTTGTTTC
PS2-10R	AGCCTCCACCCTCTGTCT
PS2-11F	TTCCATTCTGTGCACGCCTC
PS2-11R	ACCTGCCCCCACCACAATG
PS2-12F	ACACCAGGGATCACCACGCTCAC

PS2-12R	TGCCTCCTCCTCACCAAGTAAACA
APP16F	GGGTAGGCTTTGTCTTACAG
APP16R	GGCAAGACAAACAGTAGTGG
APP17F	ATAACCTCATCCAAATGTCCCC
APP17R	GTAACCCAAGCATCATGGAAGC

Table S2. Proteins measured with antibody array

ProteinName	uniprotID
EGF	P01133
G-CSF	P09919
GDNF	P39905
IL-1a	P01583
IL-3	P08700
IL-8	P10145
IL-11	P20809
MCP-3	P80098
M-CSF	P09603
MIP-1d	Q16663
TNFa	P01375
TRAIL R4	Q9UBN6
Apo (a)	P02647
ApoA2	P02652
Cathepsin D	P07339
Complement Factor H	P08603
Fibronectin	P02751
Hemoglobin	P68871
Hemopexin	P02790
IgE	P01854
IgM	P01871
Myoglobin	P02144
Neuropilin-1	O14786
SLPI	P03973
Tenascin C	P24821
Thrombospondin-4	P35443
CFXI	P03951
ACE	P12821
Amyloid beta 1-40	P05067
Amyloid beta 1-42	P05067
ANG-2	O15123
BDNF	P23560
BMP-6	P22004
BNP	P16860
CKMB	P12277
CNTF	P26441
Coagulation Factor VII	P08709
Coagulation Factor XI	P03951
Lysosomal alpha-mannosidase C peptide	O00754
FABP3	P05413
Fetuin B	Q9UGM5

Hexosaminidase A	P06865
I-309	P22362
IL-17E	Q9H293
Lumican	P51884
MIP-3a	P78556
PAPP-A	Q13219
PYY	P10082
S100b	P04271
Serpin F1	P36955
Sortilin	Q99523
Tau	P10636
TFF3	Q07654
TGF-a	P01135
tPA	P00750
TRAIL	P50591
AgRP	O00253
Axl	P30530
BLC	O43927
b-NGF	P01138
CA19-9	Q969X2
CD40 L	P29965
CEA	P06731
EGFR	P00533
Eotaxin	P51671
Eotaxin-3	Q9Y258
FGF-4	P08620
FSH	P01215
HB-EGF	Q99075
HCC-1	Q16627
HCC-4	O15467
IGF-I	P05019
IL-13	P35225
IL-16	Q14005
IL-1b	P01584
Insulin	P01308
IP-10	P02778
I-TAC	O14625
Leptin	P41159
MIP-1a	P10147
MMP-10	P09238
MMP-2	P08253
NrCAM	Q92823
NSE	P09104
PIGF	Q07326

Prolactin	P01236
PSA-Free	P55786
RAGE	Q9UQ07
Resistin	Q9HD89
SCF	P21583
TECK	O15444
Thrombomodulin	P07204
Tie-2	Q02763
TIM-1	Q96D42
uPA	P00749
VEGF-A	P15692
Cathepsin B	P07858
CHI3L1	P36222
Cystatin C	P01034
ICAM-1	P05362
PARC	P55774
PDGF-BB	P01127
RANTES	P13501
TIMP-1	P01033
VCAM-1	P19320
B2M	P61769
CRP	P02741
E-Selectin	P16581
FAP	Q12884
Ferritin	P02794
IGFBP-3	P17936
IGFBP-6	P24592
Lipocalin-2	P80188
MMP-9	P14780
NCAM-1	P13591
PAI-1	P05121
A2M	P01023
Adiponectin	Q15848
Albumin	P02768
ApoA1	P02647
ApoC1	P02654
ApoE	P02649
ApoH	P02749
Clusterin	P10909
ECM-1	Q16610
TfR	P02786
Vitronectin	P04004
vWF	P04275
A1AcidG	P02763

A1AT	P01009
AACT	P01011
ASM	P17405
Adrenomedullin	P35318
A1Micro	P02760
Apo D	P05090
BACE1	P56817
C3	P01024
Calmodulin	P0DP23
Calpain	P17655
CAMK2A	Q9UQM7
CC4	O15467
Ceruloplasmin	P00450
CFHR1	Q03591
Chromogranin A	P10645
Corticosteroid-binding globulin	P08185
CXCL16	Q9H2A7
DJ-1	Q99497
Dopamine beta-hydroxylase	P09172
Epithelial Cell Kinase	Q08345
Galanin	P22466
GOT	P17174
glycated albumin	P02768
Haptoglobin	P00738
homocysteine	Q93088
LAPA2	Q13093
Melanotransferrin	P08582
neurogranin	Q92686
NF-H	P12036
PPP	P62136
p-Tau	P10636
SAP	O75446
SOD-1	P00441
TDP-43	Q13148
Testosterone 17-beta-dehydrogenase 3	P37058
TBG	P05543
TIMP2	P16035
TIMP3	P35625
TRAF-2	Q12933
TNF-b	P01374
TTR	P02766

Table S3 The mutation status of study participants

stage	group	number	mutations
stage 1	MCI	M1	PSEN1 F105I
		M2	APP V717I
		M3	APP I716T
		M4	PSEN2 R62H
		M5	PSEN1 M139L
		M6	PSEN1 G378E
		M7	PSEN1 H163R
		M8	APP V717I
		M9	PSEN1 L282V
		M10	PSEN2 V214L
	AD	A1	PSEN1 L392V
		A2	PSEN1 H163R
		A3	PSEN1 M139V
		A4	PSEN1 G111V
		A5	PSEN1 M139L
		A6	PSEN1 F105I
		A7	APP V717I
		A8	PSEN1 H163R
		A9	APP V717I
		A10	PSEN1 P433S
stage 2	pre-MCI	P1	PSEN1 F105I
		P2	APP V717I
		P3	APP V717I
		P4	APP V717I
		P5	PSEN1 M139L
		P6	PSEN1 M139L
		P7	PSEN1 G378E
		P8	PSEN2 V214L
		P9	APP I716T
		P10	PSEN2 R62H
		P11	PSEN1 L282V
		P12	PSEN1 H163R
